# The relationship between health-promoting behaviors and negative emotions in college freshmen: a cross-lagged analysis

**DOI:** 10.3389/fpubh.2024.1348416

**Published:** 2024-04-26

**Authors:** YunFei Tao, JinLong Wu, Li Huang, KangYong Zheng, HaoWei Liu, HaoDong Tian, Li Peng

**Affiliations:** ^1^College of Physical Education, Southwest University, Chongqing, China; ^2^Department of Rehabilitation Sciences Institute, The Hong Kong Polytechnic University, Kowloon, Hong Kong SAR, China

**Keywords:** psychology, cross-lagged analysis, health-promoting behaviors, negative emotions, college students

## Abstract

**Background:**

The prevalence of mental health issues has been gradually increasing among college students in recent years. Improvements in mental health can be achieved through changes in daily behavior and the use of psychological counseling. This study aims to investigate the relationship between health-promoting behaviors and negative emotions among college freshmen as they enter the university. It also examines the impact of various sub-dimensions of health-promoting behaviors and other factors on the negative emotions (stress, anxiety, and depression) experienced by college freshmen.

**Methods:**

Using the Negative Emotion and Health-Promoting Behavior scales, a 7-month longitudinal study was conducted on 4,252 college freshmen, with collection of data at two time points (T1: November 12, 2021; T2: June 17, 2022). Out of this longitudinal study, 3,632 valid samples were obtained. This research aimed to explore the association and impact between negative emotions and the level of health-promoting behaviors among college students during their time at the university.

**Results:**

① There were significant differences in the levels of health-promoting behaviors and negative emotions over the course of 7 months (*P* < 0.05). Health-promoting behaviors were found to have a significant negative correlation with negative emotions (*P* < 0.05). ② Negative emotions at T1 significantly negatively predicted health-promoting behaviors at T2 (β = −0.11, *P* < 0.01), while health-promoting behaviors at T1 significantly negatively predicted negative emotions at T2 (β = −0.12, *P* < 0.001). ③ Stress management (β = −0.104, *P* < 0.05; β = −0.087, *P* < 0.05), self-actualization (β = −0.282, *P* < 0.01; β = −0.260, *P* < 0.05), health responsibility (β = −0.057, *P* < 0.05; β = −0.088, *P* < 0.05), and interpersonal relations (β = 0.068, *P* < 0.01; β = 0.138, *P* < 0.05) were important components in improving stress and anxiety. Self-actualization (β = −0.437, *P* < 0.001), exercise (β = 0.048, *P* < 0.001), nutrition (β = 0.044, *P* < 0.001), and interpersonal relations (β = 0.065, *P* < 0.001) were important components in improving depression. ④ Gender, place of household registration, and whether the individual is the only child were significant factors affecting negative emotions in college freshmen.

**Conclusion:**

The level of health-promoting behaviors is an important indicator for assessing the negative emotional states of college freshmen. Enhancing health-promoting behaviors across various dimensions can help alleviate different types of negative emotions. Gender, place of household registration, and being the only child are significant factors that influence negative emotions.

## 1 Introduction

In recent years, the college student population has been experiencing severe mental health issues, which is rising and attributable to unfamiliar living environments, intense academic pressures, and complex job market trends. As a result, there has been a spurt in research focusing on the psychological health issues faced by college students (1). According to statistics from the World Health Organization (WHO), over one billion people worldwide suffer from mental disorders as of June 2023, with more than one-eighth of the affected population comprising adults and adolescents. The current level of mental health services are vastly inadequate to meet the steadily increasing mental health needs. In high-income countries, only 70% of those in need can access mental health services, whereas in low-income countries, a mere 12% of the population is able to receive psychiatric treatment (2).

According to reports, the incidence of psychological disorders among Chinese university students has been rising annually and is higher than that of the general population in China. Moreover, psychological issues among Chinese students are showing a continuously increasing trend (3, 4). Although relatively severe social safety issues may occur infrequently within this group, the level of psychological problems continues to escalate (5). Chinese university students represent a unique demographic, particularly those in their 1st year, as they are at a pivotal stage transitioning from adolescence to adulthood. They encounter learning methods distinct from their high-school experiences, along with changes in diet, exercise, academic pressure in university, social interactions with peers, and uncertainties about the future. All these factors may contribute to the accumulation and even eruption of mental health issues within this group (6–9). Research indicates that negative emotions are significant indicators of mental health, primarily manifesting as a combination of anxiety, depression, and stress. However, the accumulation of negative emotions may also become a major risk factor for physical health (10). For instance, the isolation resulting from pandemic control measures has led to more severe negative emotions among college students compared to non-lockdown periods. Studies have found that during the pandemic, 22.4% of college students reported symptoms of anxiety, 35.1% reported symptoms of depression (11), and 7.2% exhibited suicidal tendencies (12). In light of these findings, given the gradual increase in the level of negative emotions among college freshmen and the significant challenge such a trend poses to public safety, it is imperative to promptly address the mental health concerns and negative emotional states of this particular group. If their psychological issues continue to be overlooked, allowing them to accumulate, it could lead to unpredictable and potentially severe consequences.

At the same time, some studies suggest that there is an extremely close relationship between the level of negative emotions in the college student population and their health-promoting behaviors (13, 14), although the specific effects are not clear. Health-promoting behaviors refer to an individual's lifestyle choices that promote health, which includes various actions such as exercise, nutrition, and others, and can be defined as “multi-dimensional, self-initiated continuous, daily activity undertaken with the deliberate aim of maintaining or enhancing the level of an individual's health, wellbeing, and self-actualization.” Research has found that college students with higher levels of physical activity tend to have lower levels of anxiety and depression (15, 16). Additionally, a higher quality of interpersonal relationships can lead to lower levels of anxiety and stress (17). Furthermore, longitudinal studies have found that the level of interpersonal relationships among college students can directly predict their levels of depression (18, 19). Additionally, there is evidence to suggest a link between diet and negative emotions. Studies have shown that poor dietary habits among college students are associated with increased levels of depression and anxiety (20, 21). This evidence underscores the potential role of health-promoting behaviors in the early prevention of mental health issues and the management of negative emotions among college students. Therefore, it is important to conduct research into the relationship between health-promoting behaviors and negative emotions to determine how different levels and aspects of these behaviors impact anxiety, depression, and stress. Such research is essential for enhancing the psychological wellbeing of college students.

With this context in mind, the primary aim of this study is to explore the relationship between negative emotions (namely, depression, anxiety, and stress) and health-promoting behaviors and its impact among new university students in China through two longitudinal surveys of freshmen (T1: November 12, 2021; T2: June 17, 2022) as they acclimate to campus life. Compared to existing research on negative emotions and mental health among college students, this study aims to longitudinally analyze the predictive impact of health-promoting behaviors on college students' negative emotions using a cross-lagged panel model. Concurrently, by employing cross-sectional regression models, it will analyze the protective factors and improvement effects of demographic variables and health-promoting behaviors, along with their subdimensions, on college students' negative emotions. This approach is intended to provide ample evidence and a theoretical basis for reducing the levels of negative emotions and improving mental health issues among college students. Building on previous research findings, this study hypothesizes that as college freshmen enhance their sense of agency, health consciousness, and health capabilities during their time at the university, their levels of health-promoting behaviors will increase, which in turn will alleviate the negative emotions associated with college life. The results of this study are expected to have significance for the prevention and improvement of psychological issues among college students and aid in exploring pathways for the prevention and treatment of mental health issues in this population.

## 2 Objects/data sources and methods

### 2.1 Objects/data sources

This study focuses on freshmen at Southwest University in Chongqing, China. Through simple random sampling, a questionnaire survey was conducted on a randomly selected sample of 4,252 freshmen from this university. The questionnaire consisted of three parts: demographic information (such as gender, age, etc.), health-promoting behaviors, and a negative emotion scale. The specific survey details are as follows: the randomly selected students completed two rounds of questionnaire surveys at the Physical Fitness Test Center of Southwest University, with a 7-month interval between the two surveys (T1: November 12, 2021; T2: June 17, 2022). The same questionnaire was used and the same batch of participants were involved in both the surveys. Based on the sample size formula, which is 20 times the number of questionnaire items (80 questions) plus 10% for invalid questionnaires, the minimum sample size for this survey was 1,760. After the test concluded, a total of 4,252 samples were obtained, which meets the minimum sample size requirement for this survey. To ensure the authenticity and reliability of the data, this study filtered the obtained data from 4,252 participants. The inclusion criteria were: (1) the questionnaire was completed in 5–15 min and (2) the participants took part in both rounds of the complete test and their student ID and name matched in both the surveys. After filtering and matching the samples, a total of 3,632 valid samples were obtained, with a sample loss of 620, resulting in an effectiveness rate of 85.42%. Among the valid samples, there were 1,340 men (36.8%) and 2,292 women (63.1%); 1,550 were the only child in the family (42.68%) and 2,082 were not the only child in the family (57.32%); 1,823 urban residents (50.19%) and 1,809 rural residents (49.81%). After screening, the average age of the students was 18.92 ± 0.50 years. This experiment was approved by the Ethics Committee of Southwest University (Approval No.: SWH202011281421), and all participants signed an informed consent form before the experiment.

### 2.2 Methods

#### 2.2.1 Negative emotion scale

The Chinese version of the Depression Anxiety Stress Scales (DASS-21) was originally developed by Lovibond et al. (22) and has since been translated into multiple languages. It is now widely used in China and has proven to be effective in measuring levels of negative emotions and its various dimensions. The Chinese version of the DASS has good reliability and validity, with the internal consistency coefficients (Cronbach's alpha) for the depression, anxiety, and stress subscales being 0.83, 0.80, and 0.82, respectively, and 0.92 for the total DASS score (23–25). The scale consists of three dimensions: anxiety, depression, and stress. Each of them have seven items, making it a total of 21 items. The questionnaire adopts a four-point scoring: “0” represents “never;” “1” represents “sometimes;” “2” represents “often;” and “3” represents “almost always.” The higher the scale score, the more serious the negative emotions. The scoring criteria are as follows: for the depression dimension, 0–9 is “normal;” 10–13 is “mild;” 14–20 is “moderate;” 21–27 is “severe;” and 28+ is “extremely severe;” for the anxiety dimension, 0–7 is “normal;” 8–9 is “mild;” 10–14 is “moderate;” 15–19 is “severe,” and 20+ is “extremely severe;” for the stress dimension, 0–14 is “normal;” 15–18 is “mild;” 19–25 is “moderate;” 26–33 is “severe;” and 34+ is “extremely severe.” In this study, the Cronbach's α-values of the two tests were 0.895 and 0.935, respectively.

#### 2.2.2 Health promotion behavior scale

The Health-Promoting Lifestyle Profile II (HPLP-II) scale was developed by Walker et al. (26), based on Pender's Health Promotion Model, and is designed to effectively measure health-promoting behaviors and their various dimensions. The Chinese version of the HPLP-II has demonstrated good reliability and validity and has been widely used for evaluating the lifestyle of university students. The Cronbach's alpha coefficients for the subscales of the questionnaire are as follows: self-actualization (0.904), health responsibility (0.814), physical activity (0.809), nutrition (0.757), interpersonal support (0.800), stress management (0.702), and for the overall Health-Promoting Lifestyle Profile (HPLP) (0.922) (26–28). The scale was a relatively mature health behavior assessment instrument at home and abroad, including self-actualization (nine items), health responsibility (nine items), physical activity (eight items), nutrition (nine items), interpersonal relations (nine items), and stress management (eight items), making it a total of 52 items. The Likert four-level scoring method (1 = never, 2 = sometimes, 3 = often, 4 = routinely) is adopted for this measure, with scores ranging from 52 to 208. The scoring is done as follows: “poor” for a score of 52–90, “general” for a score of 91–129, “good” for a score of 130–168 for, and “excellent” for a score of 169–208 for. Higher scores represented higher levels of health behavior, and each dimension within the scale could be employed independently of the other dimensions. In this study, the Cronbach's α values of the two tests were 0.932 and 0.955, respectively.

### 2.3 Statistical processing

Statistical data analysis was conducted using SPSS 25.0 and Amos 24.0 software. Initially, the data was subjected to a normality test and a common method bias test. Subsequently, a one-way analysis of variance (ANOVA) was utilized to examine if there were differences in the levels of negative emotions and health-promoting behaviors between the two measurement points. The effect size, variance among groups, and significance level were represented by η*p2, F*, and *p*-values, respectively. Additionally, correlation analysis was employed to investigate the relationship between negative emotions and health-promoting behaviors across the different time points.

A structural equation model was constructed using Amos24.0 to analyze and verify the cross-lagged model. The χ^2^ statistical index and the root mean square error of approximation (RMSEA) were utilized as the absolute fitting measures. Incremental fit index (IFI), Tucker–Lewis index (TLI), and goodness of fit index (GFI) were used as incremental fit indexes. χ^2^/df < 5, RMSEA < 0.08, IFI, TLI, and GFI values > 0.9 indicated that the model fitted well. Finally, a generalized linear regression model was established with depression, anxiety, and stress symptoms as the dependent variables and health behavior factors significantly related to one or more symptoms as independent variables. The data were presented in the form of mean plus or minus standard deviation (M ± SD). The significance level of statistical analysis was set as *p* < 0.05 for statistical difference, *p* < 0.01 for significant statistical difference, and *p* < 0.001 for the extremely significant statistical difference.

## 3 Results

### 3.1 Sample randomization test and common method bias

Due to the use of questionnaire surveys to assess the levels of negative emotions and health-promoting behaviors among college freshmen, the selected sample may have errors compared to the original sample. Therefore, through the analysis and testing of the sample, it was found that there were no significant differences between the selected sample and the original sample in terms of the levels of negative emotions and health-promoting behaviors, as well as their demographic factors (*P* > 0.05). As this study adopted self-reported data, there may be common method bias. This study took advantage of confirmatory factor analysis in Amos to test the common method bias of all self-evaluation items. The results showed that the model fitting was poor (χ^2^/df = 407.03, CFI = 0.441, GFI = 0.424, AGFI = 0.270, NFI = 0.440, RMSEA = 0.263). The original model was χ^2^/df = 43.78, CFI = 0.943, GFI = 0.884, AGFI = 0.848, NFI = 0.942, RMSEA = 0.085, so there was no common method bias in the study.

### 3.2 Correlation analysis of negative emotions and health behavior among college students

Correlation analysis between negative emotions and health-promoting behaviors across the two measurements is presented in Table 1. The results of the two measurements indicated that health-promoting behaviors and negative emotions were correlated with each other. The correlation for negative emotions was significant with *r* = 0.316 (*p* < 0.001), and for health-promoting behaviors, it was also significant, with *r* = 0.295 (*p* < 0.001). Health-promoting behaviors were negatively correlated with negative emotions, with significant correlations at T1 and T2 for negative emotions and health behaviors at *r* = −0.032 (*p* < 0.001) and *r* = −0.371 (*p* < 0.001), respectively. The correlation between negative emotions at T1 and health-promoting behaviors at T2 was significant at *r* = −0.108 (*p* < 0.001), and the correlation between negative emotions at T2 and health-promoting behaviors at T1 was significant at *r* = −0.136 (*p* < 0.001). The correlation between negative emotions and health behaviors was significant, meeting the prerequisite conditions for cross-lagged analysis.

**Table 1:** Correlation analysis of negative emotions and health behavior.

		**Negative emotions**		**Health behavior**	
**Variables**	**Time points**	**T1**	**T2**	**T1**	**T2**
Negative emotions	T1	1	0.316^***^	−0.032^*^	−0.108^***^
	T2		1	−0.136^***^	−0.371^***^
Health behavior	T1			1	0.295^***^
	T2				1

^*^P < 0.05, ^**^P < 0.01, ^***^P < 0.001.

### 3.3 Differences in negative emotions and health behavior among college students at T1 and T2

In this study, negative emotions and three dimensions, health behavior and six dimensions, and gender were used as dependent variables, and measurement time T1 and T2 were used as the factors. One-way analysis of variance was performed on the data at two measurement time points. The results showed that the total scores of negative emotions (*F* = 21.18, *P* < 0.001), anxiety (*F* = 43.13, *P* < 0.001), depression (*F* = 31.68, *P* < 0.001), stress (*F* = 33.91, *P* < 0.001), health promotion behavior (*F* = 14.36, *P* < 0.001), self-realization (*F* = 13.64, *P* < 0.001), physical activity (*F* = 14.64, *P* < 0.001), nutrition (*F* = 13.84, *P* < 0.001), interpersonal relations (*F* = 13.61, *P* < 0.001), stress management (*F* = 19.11, *P* < 0.001), and health responsibility (*F* = 5.43, *P* < 0.001) had significant main effects on time (see Table 2). The above results indicated that college students showed distinct differences in their total and individual dimension scores of negative emotions and health behavior in both tests during the pandemic period when controls were in place. Specific analysis of each dimension showed a significant improvement in overall negative emotions (Δ = −1.02) over time and a sharp decrease in both anxiety (Δ = −1.12) and stress (Δ = −0.1), but an increasing tendency for depression instead (Δ = 0.18). The overall health behavior (Δ = −1.31) decreased significantly, with noteworthy decreases in the dimensions of self-actualization (Δ = −0.98), interpersonal relations (−0.97), and stress management (−0.09) and an increasing trend in the dimensions of nutrition (Δ = 0.03) and health responsibility (Δ = 0.72).

**Table 2:** One-way ANOVA (*M* ± *SD*) analysis for each dimension of negative emotions and health behavior.

**Dimensions**	**T1**	**T2**	***ηp2***	***F***	***P***	**Differential value**
Negative emotions	31.43 ± 8.79	30.41 ± 8.88	0.210	21.18	0.000	−1.02
Anxiety	11.05 ± 3.30	9.93 ± 2.88	0.152	43.13	0.000	−1.12
Depression	9.72 ± 2.90	9.9 ± 3.14	0.143	31.68	0.000	0.18
Stress	10.67 ± 3.45	10.57 ± 3.39	0.145	33.91	0.000	−0.10
Health behavior	132.49 ± 22.09	131.18 ± 23.09	0.172	14.36	0.000	−1.31
Self-actualization	24.61 ± 4.77	23.63 ± 4.60	0.093	13.64	0.000	−0.98
Health responsibility	21.03 ± 4.49	21.75 ± 4.80	0.097	14.64	0.000	0.72
Physical activity	19.34 ± 4.06	19.34 ± 4.05	0.089	13.84	0.000	0.00
Nutrition	22.11 ± 4.00	22.14 ± 4.15	0.094	13.61	0.000	0.03
Interpersonal relations	24.9 ± 4.29	23.93 ± 4.37	0.089	19.11	0.000	−0.97
Stress management	20.49 ± 3.88	20.4 ± 3.84	0.113	5.43	0.000	−0.09

### 3.4 The relationship between negative emotions and health behavior

**Figure 1:**
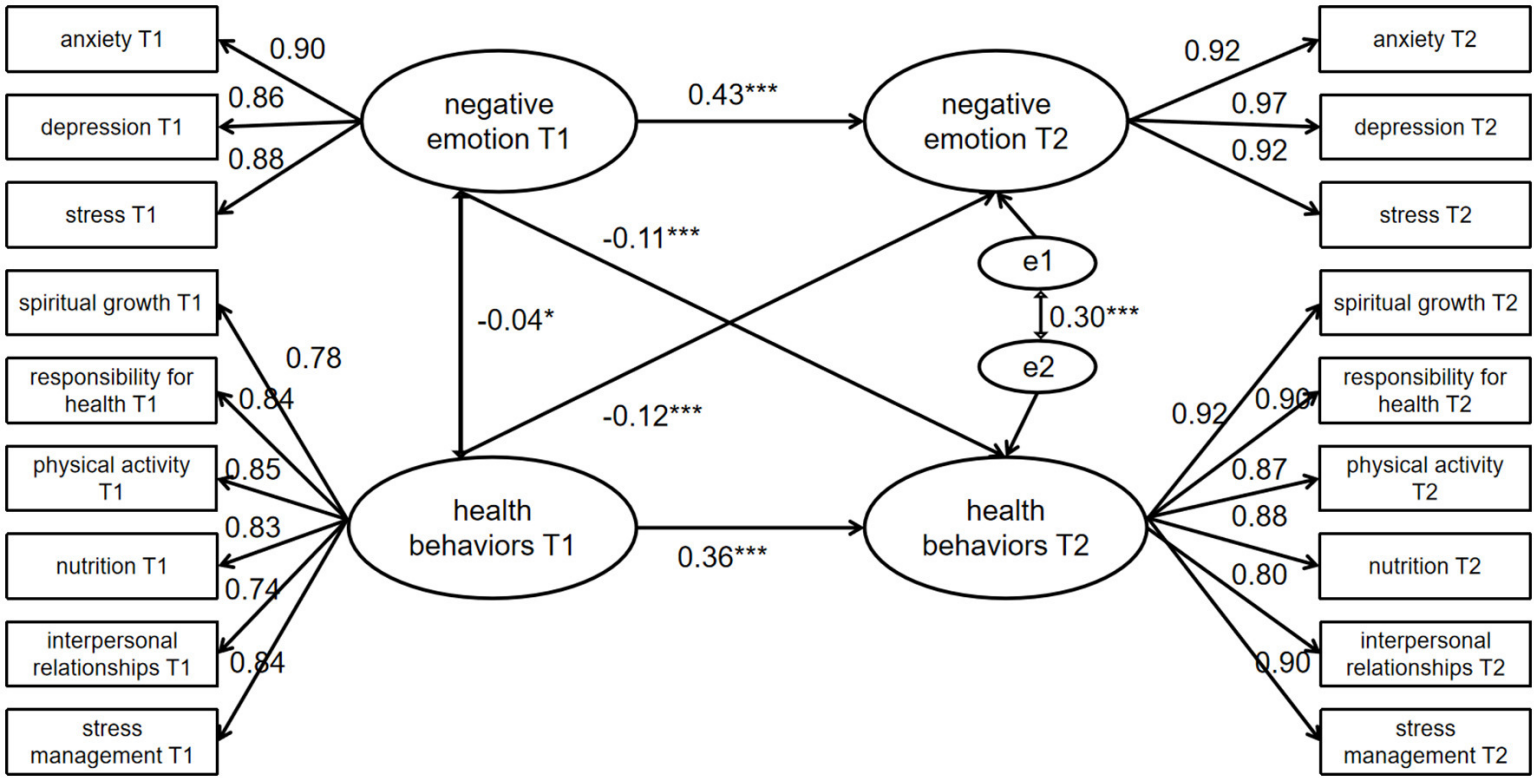
The cross-lagged analysis of negative emotions and health behavior. ^*^P < 0.05, ^**^P < 0.01, ^***^P < 0.001.

After establishing a significant correlation between negative emotions and health behaviors, this study used a cross-lagged model to analyze the data from two assessments within a year, examining if there is a bidirectional effect between negative emotions and health-promoting behaviors. The model was constructed using the three dimensions of negative emotions—stress, depression, and anxiety—and the six dimensions of the health-promoting behaviors scale—stress management, nutrition, exercise, self-actualization, interpersonal relationships, and health responsibility—as manifest variables. Before conducting the cross-lagged panel model analysis, autoregressive and single cross-lagged models were established to verify the stability of the cross-lagged model. Specific path coefficients and their significance are shown in Table 3, and all paths' fit indices met the standards (χ^2^/df < 5, RMSEA < 0.08, IFI, TLI, and GFI values > 0.9). After validating the autoregressive and single cross-lagged models, a double cross-lagged model was established. The specific model is shown in Figure 1. To simplify the model, latent variable residuals and predictive paths between latent variables are not displayed in the figure. The model was constructed using Amos 24.0 and examined for fit using maximum likelihood estimation. The results indicated that the fit indices for each indicator were good (χ^2^/df = 4.010, CFI = 0.995, NFI = 0.993, TLI = 0.991, RFI = 0.989, RMSEA = 0.023). From the cross-lagged path diagram, it can be seen that the autoregressive coefficients for negative emotions and health-promoting behaviors at the two measurement time points were stable and highly significant, with β = 0.43 (*P* < 0.001) and β = 0.36 (*P* < 0.001), respectively. At both T1 (β = −0.04, *P* < 0.05) and T2 (β = −0.30, *P* < 0.001) time points, there was a negative correlation between negative emotions and health-promoting behaviors. After controlling for the autoregression of negative emotions and health behaviors, as well as the correlation between the two variables at the same measurement time point, the results showed that negative emotions measured at T1 significantly negatively predicted health-promoting behaviors at T2 (β = −0.11, *P* < 0.01). Similarly, health-promoting behaviors measured at T1 significantly negatively predicted negative emotions at T2 (β = −0.12, *P* < 0.001). The results indicate that there is a mutual negative influence between negative emotions and health-promoting behaviors.

**Table 3:** Overview of the standardized stability and cross-lagged coefficients.

**Model**	**Autoregressive path**	**β**	**Cross-lagged path**	**β**
	**Autoregressive model**			
Model 1	(HPLP-II) T1-(HPLP-II) T2	0.450^***^		
	(Negative emotions) T1-(Negative emotions) T2	0.392^***^		
	**Single lagged dependent variable model**			
Model 2	(HPLP-II) T1-(HPLP-II) T2	0.472^***^	(HPLP-II) T1-(Negative emotions) T1	−0.476^***^
	(Negative emotions) T1-(Negative emotions) T2	0.352^***^	(HPLP-II) T1-(Negative emotions) T2	−0.096^***^
Model 3	(Negative emotions) T1-(HPLP-II) T1	−0.480^***^	(HPLP-II) T1-(HPLP-II) T2	0.419^***^
	(Negative emotions) T1-(HPLP-II) T2	−0.161^***^	(Negative emotions) T1-(Negative emotions) T2	0.382^***^
	**Double lagged dependent variable Model**			
Model 4	(HPLP-II) T1-(HPLP-II) T2	0.362^***^	(HPLP-II) T1-(Negative emotions) T1	−0.432^***^
	(Negative emotions) T1-(Negative emotions) T2	0.428^***^	(HPLP-II) T1-(Negative emotions) T2	−0.095^***^
			(Negative emotions) T1-(HPLP-II) T1	−0.432^***^
			(Negative emotions) T1-(HPLP-II) T2	−0.159^***^

^*^P < 0.05, ^**^P < 0.01, ^***^P < 0.001.

### 3.5 Protective factors for college students to reduce negative emotions

As indicated in Table 4, generalized linear regression analysis was performed after including demographic factors such as gender, age, place of household registration, and being the only child as covariates. This analysis aimed to assess the impact of the subdimensions of health-promoting behaviors (self-actualization, health responsibility, exercise, nutrition, interpersonal relations, and stress management) on the levels of anxiety, depression, and stress among college freshmen. The results indicated that stress management, self-actualization, and health responsibility all had a negative effect on the stress and anxiety levels of college freshmen, while interpersonal relations had a positive effect on these levels. Specifically, higher levels of stress management (β = −0.104, *P* < 0.05; β = −0.087, *P* < 0.05), self-actualization (β = −0.282, *P* < 0.01; β = −0.260, *P* < 0.05), and health responsibility (β = −0.057, *P* < 0.05; β = −0.088, *P* < 0.05) were associated with lower levels of stress and anxiety among college freshmen. On the other hand, a higher level of interpersonal relations (β = 0.068, *P* < 0.01; β = 0.138, *P* < 0.05) was associated with higher levels of stress and anxiety. Furthermore, self-actualization was found to negatively impact the depression levels of college freshmen. In contrast, exercise, nutrition, and interpersonal relations were found to positively affect these levels. Specifically, higher self-actualization scores (β = −0.437, *P* < 0.001) were associated with lower levels of depression among college freshmen. Conversely, higher levels of exercise (β = 0.048, *P* < 0.001), nutrition (β = 0.044, *P* < 0.001), and interpersonal relations (β = 0.065, *P* < 0.001) were associated with higher levels of depression. In summary, stress management, self-actualization, health responsibility, and interpersonal relations are important predictive factors affecting the stress and anxiety levels of college freshmen, while self-actualization, exercise, nutrition, and interpersonal relations are important protective factors influencing their depression levels. Additionally, the results for demographic covariates indicated that gender, place of household registration, and whether one is the only child are also major factors influencing the stress, anxiety, and depression levels of college freshmen.

**Table 4:** Generalized linear model analysis of negative emotions and health behavior.

	**Stress**			**Anxiety**			**Depression**		
	β	***P***	***[95% CI]***	β	***P***	***[95% CI]***	β	***P***	***[95% CI]***
Stress management	−0.104	0.000^***^	−0.157, −0.052	−0.087	0.001^**^	−0.136, −0.037	0.025	0.283	−0.021, 0.071
Self-actualization	−0.282	0.000^***^	−0.327, −0.237	−0.260	0.000^***^	−0.303, −0.218	−0.437	0.000^***^	−0.476, −0.398
Health responsibility	−0.057	0.006^**^	−0.098, −0.016	−0.088	0.000^***^	−0.126, −0.050	−0.032	0.067	−0.068, 0.002
Physical activity	0.035	0.099	−0.007, 0.077	0.004	0.854	−0.036, 0.043	0.048	0.010^*^	0.011, 0.084
Nutrition	0.032	0.121	−0.008, 0.073	0.008	0.664	−0.030, 0.047	0.044	0.013^*^	0.009, 0.080
Interpersonal relations	0.068	0.003^**^	0.023, 0.114	0.138	0.000^***^	0.095, 0.182	0.065	0.002^**^	0.025, 0.105
Gender	0.897	0.000^***^	0.683, 1.111	0.718	0.000^***^	0.516, 0.920	0.554	0.000^***^	0.367, 0.740
Age	0.002	0.924	−0.048, 0.053	0.006	0.818	−0.042, 0.053	0.022	0.317	−0.021, 0.066
Household registration	−0.354	0.001^**^	−0.571, −0.136	−0.342	0.001^**^	−0.548, −0.137	−0.366	0.000^***^	−0.555, −0.177
Only child	0.254	0.023^*^	0.034, 0.473	0.275	0.009^**^	0.069, 0.482	0.242	0.013^*^	0.052, 0.433
History of smoking and drinking	0.088	0.838	−0.758, 0.934	−0.223	0.584	−1.020, 0.574	0.371	0.323	−0.365, 1.107

^*^P < 0.05, ^**^P < 0.01, ^***^P < 0.001.

## 4 Discussion

This study examined the longitudinal predictive relationship between negative emotions and health-promoting behaviors among college freshmen, as well as the cross-sectional impact of health-promoting behaviors and demographic factors on negative emotions, by employing cross-lagged and generalized linear models. The main findings revealed that over the course of 7 months, there were significant changes in the levels of negative emotions and health-promoting behaviors and their subdimensions. Specifically, the assessed levels of negative emotions and their anxiety dimension, as well as health-promoting behaviors and their subdimensions of self-actualization, interpersonal relationships, and stress management, significantly decreased. The results of the cross-lagged model indicated that there is a negative longitudinal bidirectional relationship between college freshmen's negative emotions and their levels of health-promoting behaviors. The generalized linear regression results demonstrated that within health-promoting behaviors, stress management, self-actualization, health responsibility, and interpersonal relations, as well as demographic factors such as gender, place of household registration, and whether one is the only child, are all significant predictive factors for stress and anxiety in college freshmen. Furthermore, self-actualization, exercise, nutrition, interpersonal relations, and demographic factors such as gender, place of household registration, and only-child status are also significant protective factors against depression in college freshmen.

Our research found that there were significant differences in the levels of negative emotions and health-promoting behaviors, as well as their subdimensions, among college students at T1 and T2, with both levels decreasing over time. It should be noted that as time progresses, one of the potential factors contributing to the reduction in negative emotions among college freshmen could be their growing familiarity with college life. However, this is not the only influencing factor, as during this period, no interventions were conducted on the participants. The analysis was based on the assessment of the levels of health behavior factors. Therefore, it is likely that other exposure factors, such as sleep quality, could have an impact on negative emotions. It is important to note that the complexity of human emotions and behaviors is influenced by a multifaceted array of factors, not just those measured or observed in a given study. During the second measurement of negative emotions among college freshmen, it was observed that only the level of depressive emotions showed a significant increase. This suggests that within the spectrum of negative emotions experienced by college freshmen, particular attention should be paid to the level of depression. The inference drawn from the second assessment of college freshmen suggests that the increase in the level of depression may be associated with a decrease in the levels of interpersonal relations, self-actualization, and stress management. This implies that these factors could potentially be key areas for intervention to mitigate depression among college students. To further explore the factors influencing the level of depressive emotions, this study also conducted a related analysis using generalized linear regression. The research found that self-actualization, exercise, nutrition, interpersonal relations, gender, place of household registration, and whether one is the only child are all significant protective factors against depression in college freshmen. This substantiates the previous inference and aligns with existing research, indicating that the level of depression among college students is associated with communication with peers and teachers, setting goals for self-actualization (29, 30), and self-management of academic stress (31–33). This suggests that, compared to other negative emotions and mental health issues (such as anxiety and stress) experienced by college freshmen, depression appears to be a more severe issue and requires more attention. Concurrently, the results from the correlational analysis revealed a significant negative relationship between negative emotions and the level of health-promoting behaviors. This finding is consistent with the majority of results from previous studies (34–36), which indicate that certain health behaviors (such as exercise and nutrition) are negatively correlated with negative emotions. This suggests that engaging in healthy behaviors can potentially reduce the occurrence or intensity of negative emotions such as depression, anxiety, and stress among college students.

In previous research, studies investigating the impact of health-promoting behaviors on the levels of negative emotions among college students have been relatively scarce. To further explore the longitudinal predictive relationship between these two major factors, a cross-lagged model was employed for analysis. The results showed that there is a bidirectional negative predictive relationship between negative emotions and health-promoting behaviors in college freshmen. This is consistent with the findings from researchers like Liu et al., Cao et al., and Zhang et al. (9, 36, 37). They found that higher levels of self-efficacy, physical activity, and self-control contribute to reducing the accumulation of negative emotions such as depression, releasing stress, and stabilizing mood. In addition, our study identified important protective factors for improving stress, depression, and anxiety, providing significant evidence for predicting mental health levels in college freshmen. The research has revealed that if negative emotions among current college freshmen are not alleviated and addressed in a timely manner, they will continue to accumulate, leading to a reduction in health-promoting behaviors, which in turn can cause a further accumulation of more severe negative emotions. However, since an increase in health-promoting behaviors can improve negative emotions in college freshmen, maintaining a certain level of such behaviors can serve as a preventative measure against emotional outbursts and reduce the buildup of negative emotions. In fact, there have been studies based on the health promotion model that explain the connection with negative emotions, suggesting that when people engage in behaviors that are detrimental to their health, it could lead to the onset of psychological issues, while the converse could contribute to the improvement of such issues (30, 38, 39). Building on this theoretical foundation, our study explored the relationship between the psychology and daily behaviors (health-promoting behaviors) of college freshmen and further analyzed the protective factors against negative emotions.

Through the analysis using a generalized linear regression model, it was observed that within the subdimensions of health-promoting behaviors, lower levels of stress management, self-actualization, and health responsibility behaviors were associated with higher levels of stress and anxiety. Conversely, an increase in interpersonal relationship behaviors was found to help reduce stress and anxiety. In the study of depression, it was observed that more self-actualization behaviors could increase depressive emotions. However, an increase in nutrition, exercise, and interpersonal relationship behaviors could effectively reduce depression. This is similar to previous studies which suggested that adequate nutrition, exercise, and interpersonal relationships are one of the effective ways to improve and protect students' mental health (16, 36). However, the difference lies in that these studies seemed to focus only on individual factors, such as the impact of physical activity, diet, or mindfulness, with only a few discussing the interactive effects of these factors on negative emotions. Among demographic factors, gender, household registration location (urban vs. rural), and whether an individual is the only child were identified as significant factors affecting negative emotions, with large regression coefficients. This is consistent with existing research which indicates that being women, from a rural area, and not being the only child can lead to higher levels of negative emotions (12, 40, 41).

In summary, this study analyzed the negative predictive relationship between negative emotions and health-promoting behaviors among college freshmen through both longitudinal and cross-sectional research. It also delved into the effects of subdimensions of health-promoting behaviors and demographic factors on the stress, anxiety, and depression experienced by college freshmen. This research has practical significance for the prevention and improvement of negative emotions among college students. At the school and societal levels, the role of healthy behaviors in moderating the emotions of college freshmen can be further emphasized to help students understand the practical importance of health-promoting behaviors, encouraging and monitoring the increase of such behaviors to alleviate negative emotions and prevent significant public safety issues. From a government macro-control perspective, reducing the rural–urban divide, advocating for gender equality, and liberalizing birth policies may help to reduce levels of stress, anxiety, and depression among the college student population.

## 5 Advantages and limitations

This study assessed the health-promoting behaviors and levels of negative emotions among college freshmen using the Health-Promoting Lifestyle Profile II (HPLP-II) and Depression Anxiety Stress Scales (DASS-21) scales. A cross-lagged model was employed to explore the impact of health-promoting behaviors and their subdimensions on negative emotions. Additionally, a generalized linear model was used to investigate the protective factors of health-promoting behaviors and their subdimensions against negative emotions. The goal was to identify predictive factors of daily health behavior suitable for reducing negative emotions and alleviating mental health issues among college freshmen. These findings play an important role in protecting and preventing psychological health issues in this population.

In addition to its strengths, this study also has some limitations. For instance, while health-promoting behaviors encompass most aspects of college freshmen's lives, and we have accounted for the regression impact of demographic variables on negative emotions through covariates, it is important to acknowledge that there are many other exposure factors that may affect negative emotions. These include family stress, financial pressure, friendly relationships between the sexes, and even factors such as weather, all of which require extensive research to infer and analyze their impact on negative emotions. Secondly, this study only included two time points, and the 7-month period may not be representative of the entire academic life of 1st-year college students (although this possibility is small). Future studies could consider including more measurement time points and shorter intervals, which would likely reduce research errors. Finally, the sample of over 3,000 students in this study may not be perfectly representative of the national population, which could introduce a certain degree of error in terms of representativeness. Based on this, future research will require more studies from different regions to validate these results.

## 6 Conclusions

College freshmen's negative emotions and health-promoting behaviors are significantly negatively correlated. The longitudinal analysis indicates that initial negative emotions and health-promoting behaviors can significantly negatively predict subsequent levels of negative emotions. Within health-promoting behaviors, stress management, self-actualization, health responsibility, and interpersonal relationship dimensions are significant protective factors against stress and anxiety. Self-actualization, exercise, nutrition, and interpersonal relationships are significant protective factors against depressive emotions. Gender, household registration location, and whether one is the only child are significant factors affecting the negative emotions of college freshmen.

## Data availability statement

The original contributions presented in the study are included in the article/supplementary material, further inquiries can be directed to the corresponding author.

## Ethics statement

The studies involving humans were approved by Ethics Review Committee of Southwest University Hospital, Chongqing, China. The studies were conducted in accordance with the local legislation and institutional requirements. The participants provided their written informed consent to participate in this study. Written informed consent was obtained from the individual(s) for the publication of any potentially identifiable images or data included in this article.

## Author contributions

YT: Conceptualization, Data curation, Formal analysis, Investigation, Writing – original draft, Writing – review & editing. JW: Data curation, Writing – review & editing. KZ: Supervision, Writing – review & editing. LH: Conceptualization, Data curation, Supervision, Writing – review & editing. HL: Data curation, Supervision, Writing – review & editing. HT: Data curation, Supervision, Writing – review & editing. LP: Funding acquisition, Writing – review & editing.

## References

[1] Beiter R Nash R McCrady M Rhoades D Linscomb M Clarahan M. The prevalence and correlates of depression, anxiety, and stress in a sample of college students. J Affect Disord. (2015) 173:90–6. 10.1016/j.jad.2014.10.054 25462401

[2] Cuijpers P Javed A Bhui K. The WHO World Mental Health Report: a call for action. Br J Psychiatr. (2023) 222:227–9. 10.1192/bjp.2023.9 36794529

[3] Jin Z Cao W Wang K Meng X Shen J Guo Y. Mental health and risky sexual behaviors among Chinese college students: a large cross-sectional study. J Affect Disord. (2021) 287:293–300. 10.1016/j.jad.2021.03.067 33812242

[4] Zhang H Zhao H. Changes in Chinese adolescent college students' psychological security during 2004-2020: a cross-temporal meta-analysis. J Adolesc. (2023) 95:631–46. 10.1002/jad.12147 36751136

[5] Liu X Zhang Y Luo Y. Does subjective well-being improve self-rated health from undergraduate studies to three years after graduation in China? Healthcare. (2023) 11:2813. 10.3390/healthcare11212813 37957958 PMC10649333

[6] Guo C Cui Y Xia Z Hu J Xue Y Huang X. Association between health literacy, depressive symptoms, and suicide-related outcomes in adolescents: a longitudinal study. J Affect Disord. (2023) 327:15–22. 10.1016/j.jad.2023.01.054 36707037

[7] Cheng S An D Yao Z Liu JJ Ning X Wong JP. Association between mental health knowledge level and depressive symptoms among Chinese College Students. Int J Environ Res Public Health. (2021) 18:1850. 10.3390/ijerph18041850 33672872 PMC7918134

[8] Liu X Zhang Y Gao W Gao X. Developmental trajectories of depression, anxiety, and stress among college students: a piecewise growth mixture model analysis. Human Soc Sci Commun. (2023) 10:1–10. 10.1057/s41599-023-02252-2

[9] Liu X Li Y Cao X. Bidirectional reduction effects of perceived stress and general self-efficacy among college students: a cross-lagged study. Human Soc Sci Commun. (2024) 11:1–8. 10.1057/s41599-024-02785-0

[10] Puustinen PJ. Screening for Psychological Distress. (2012). Available online at: http://www.researchgate.net/publication/286847943_Screening_for_psychological_distress (accessed March 18, 2024).

[11] Kalkbrenner MT Flinn RE Sullivan DK Arteaga LEE. A mental health literacy approach to supporting first-generation community college student mental health: the REDFLAGS model. Commun Coll Rev. (2021) 49:243–61. 10.1177/00915521211002893

[12] Chen R-N Liang S-W Peng Y Li X-G Chen J-B Tang S-Y. Mental health status and change in living rhythms among college students in China during the COVID-19 pandemic: a large-scale survey. J Psychosom Res. (2020) 137:110219. 10.1016/j.jpsychores.2020.110219 32862063 PMC7428432

[13] Lee RLT Loke AJTY. Health-promoting behaviors and psychosocial wellbeing of university students in Hong Kong. Publ Health Nurs. (2005) 22:209–20. 10.1111/j.0737-1209.2005.220304.x 15982194

[14] Cao X Ji S. Bidirectional relationship between self-rated health and the big five personality traits among Chinese adolescents: a two-wave cross-lagged study. Human Soc Sci Commun. (2024) 11:1–11. 10.1057/s41599-024-02699-x

[15] Deforche B Van Dyck D Deliens T De Bourdeaudhuij I. Changes in weight, physical activity, sedentary behaviour and dietary intake during the transition to higher education: a prospective study. Int J Behav Nutr Phys Act. (2015) 12:16. 10.1186/s12966-015-0173-9 25881147 PMC4332914

[16] Chi G Wang L. The association of sports participation with depressive symptoms and anxiety disorder in adolescents. Front Publ Health. (2022) 10:860994. 10.3389/fpubh.2022.860994 35719630 PMC9203890

[17] Meng T He Y Zhang Q Yu F Zhao L Zhang S. Analysis of features of social anxiety and exploring the relationship between childhood major adverse experiences and social anxiety in early adulthood among Chinese college students. J Affect Disord. (2021) 292:614–22. 10.1016/j.jad.2021.05.105 34153832

[18] Wang W Xu H Li S Jiang Z Sun Y Wan Y. The impact of problematic mobile phone use and the number of close friends on depression and anxiety symptoms among college students. Front Psychiatry. (2023) 14:1281847. 10.3389/fpsyt.2023.1281847 38260802 PMC10800545

[19] Frazier P Liu Y Asplund A Meredith L Nguyen-Feng VN. US college student mental health and COVID-19: comparing pre-pandemic and pandemic timepoints. J Am Coll Health. (2023) 71:2686–96. 10.1080/07448481.2021.1987247 34762560

[20] Keck MM Vivier H Cassisi JE Dvorak RD Dunn ME Neer SM. Examining the role of anxiety and depression in dietary choices among college students. Nutrients. (2020) 12:2061. 10.3390/nu12072061 32664465 PMC7400947

[21] Chi X Liang K Chen S-T Huang Q Huang L Yu Q. Mental health problems among Chinese adolescents during the COVID-19: The importance of nutrition and physical activity. Int J Clin Health Psychol. (2021) 21:100218. 10.1016/j.ijchp.2020.100218 33391373 PMC7759093

[22] Lovibond SH Lovibond PF. Depression anxiety stress scales. Psychol Asses. (1995). 10.1037/t01004-000 7726811

[23] Lovibond SH Lovibond PF. Manual for the Depression Anxiety Stress Scales. (2002). Available online at: http://www.mendeley.com/catalog/manual-depression-anxiety-stress-scales/ (accessed March 18, 2024).

[24] Nanthakumar S Bucks RS Skinner TC Starkstein S Hillman D James A. Assessment of the Depression, Anxiety, and Stress Scale (DASS-21) in untreated obstructive sleep apnea (OSA). Psychol Assess. (2017) 29:401. 10.1037/pas0000401 27936819

[25] Chan RCK Xu T Huang J Wang Y Zhao Q Shum DHK. Extending the utility of the Depression Anxiety Stress scale by examining its psychometric properties in Chinese settings. Psychiatry Res. (2012) 200:879–83. 10.1016/j.psychres.2012.06.041 22921506

[26] Walker SN Sechrist KR Pender NJ. The health-promoting lifestyle profile: development and psychometric characteristics. Nurs Res. (1987) 36:76–81. 10.1097/00006199-198703000-00002 3644262

[27] Scholten S Velten J Bieda A Zhang XC Margraf J. Testing measurement invariance of the Depression, Anxiety, and Stress Scales (DASS-21) across four countries. Psychol Assess. (2017) 29:440. 10.1037/pas0000440 28125249

[28] Wang K Shi HS Geng FL Zou LQ Tan SP Wang Y. Cross-cultural validation of the Depression Anxiety Stress Scale-21 in China. Psychol Assess. (2015) 28:e88. 10.1037/pas0000207 26619091

[29] Kalpidou M Costin D Morris J. The relationship between Facebook and the wellbeing of undergraduate college students. Cyberpsychol Behav Soc Netstudy. (2011) 14:183–9. 10.1089/cyber.2010.0061 21192765

[30] Shi X Wang A Zhu Y. Longitudinal associations among smartphone addiction, loneliness, and depressive symptoms in college students: disentangling between- and within-person associations. Addict Behav. (2023) 142:107676. 10.1016/j.addbeh.2023.107676 36878182

[31] Yeh PM Moxham L Patterson C Antoniou C Liou JC. A comparison of psychological well-being, coping strategies, and emotional problems between Taiwanese and Australian Nursing Students. J Nurs Res. (2023) 31:e264. 10.1097/jnr.0000000000000543 36826356

[32] Yang SY Fu SH Chen KL Hsieh PL Lin PH. Relationships between depression, health-related behaviors, and internet addiction in men women junior college students. PLoS ONE. (2019) 14:e0220784. 10.1371/journal.pone.0220784 31398212 PMC6688785

[33] Steinhardt M Dolbier C. Evaluation of a resilience intervention to enhance coping strategies and protective factors and decrease symptomatology. J Am Coll Health. (2008) 56:445–53. 10.3200/JACH.56.44.445-454 18316290

[34] Hou T Xie Y Mao X Liu Y Zhang J Wen J. The mediating role of loneliness between social support and depressive symptoms among Chinese rural adolescents during COVID-19 outbreak: a comparative study between left-behind and non-left-behind students. Front Psychiatry. (2021) 12:740094. 10.3389/fpsyt.2021.740094 34497549 PMC8420998

[35] Rezapour M. Factors associated with subjective state of health in college students. Front Psychol. (2022) 13:985982. 10.3389/fpsyg.2022.985982 36312060 PMC9613111

[36] Cao X Zhang Q Liu X. Cross-lagged relationship between physical activity time, openness and depression symptoms among adolescents: evidence from China. Int J Mental Health Promot. (2023) 25:1009–18. 10.32604/ijmhp.2023.02936537303558

[37] Zhang Q Wang X Miao L He L Wang H. The effect of chronotype on risk-taking behavior: the chain mediation role of self-control and emotional stability. Int J Environ Res Public Health. (2022) 19:16068. 10.3390/ijerph192316068 36498142 PMC9737074

[38] Copeland W E Mcginnis E Bai Y Adams Z Nardone H Devadanam V. Impact of COVID-19 pandemic on college student mental health and wellness. J Am Acad Child Adolesc Psychiatr. (2021) 60:134–41.e2. 10.1016/j.jaac.2020.08.466 33091568 PMC8173277

[39] Zhang X Shi X Wang Y Jing H Zhai Q Li K. Risk factors of psychological responses of Chinese university students during the COVID-19 outbreak: cross-sectional web-based survey study. J Med Internet Res. (2021) 23:e29312. 10.2196/29312 34156961 PMC8297601

[40] Lin J Zou L Lin W Becker B Yeung A Cuijpers P. Does gender role explain a high risk of depression? A meta-analytic review of 40 years of evidence. J Affect Disord. (2021) 294:261–78. 10.1016/j.jad.2021.07.018 34304081

[41] Chen J. Hysteresis effects and emotional suffering: Chinese rural students' first encounters with the urban university. Sociol Res Onl. (2022) 27:101–17. 10.1177/1360780420949884

